# Delineation of two new, highly similar species of Taiwanese *Cylindera* tiger beetles (Coleoptera, Carabidae, Cicindelinae) based on morphological and molecular evidence

**DOI:** 10.3897/zookeys.875.37856

**Published:** 2019-09-11

**Authors:** Ming-Hsun Chou, Wen-Bin Yeh

**Affiliations:** 1 Department of Entomology, National Chung Hsing University, 250 Kuo Kuang Road, Taichung 40227, Taiwan National Chung Hsing University Taichung Taiwan

**Keywords:** COI, key, new species, taxonomy, 16S rDNA, 28S rDNA

## Abstract

Tiger beetles have been recognized primarily based on morphological characters. However, the variations of elytral maculation and coloration sometimes lead to misjudgment in species classification and the overlooking of the existence of cryptic species. Recently, specimens of two endemic species of Taiwanese *Cylindera*, *C. sauteri* and *C. pseudocylindriformis*, exhibit morphologically recognizable forms, indicating that some undescribed species may exist. To clarify their taxonomic status, morphological characteristics including male genitalia were examined and two mitochondrial genes, COI and 16S rDNA, and one nuclear 28S rDNA were analyzed. Molecular phylogenetic inferences indicated that both forms in both species are reciprocally monophyletic. Moreover, molecular dating showed the forms diverged approximately 1.3 million years ago. Two new species, *Cylindera ooa***sp. nov.** and *Cylindera autumnalis***sp. nov.**, are thereby described. The main recognizable characteristics separating *C. ooa***sp. nov.** from *C. sauteri* are the lack of a triangular spot at the middle edge of elytron and the elongated but not rounded subapical spot. For *C. autumnalis***sp. nov.**, the apical lunula near the elytral suture is thickened but not linear and slender, and its elytra are more metallic brownish than those of *C. pseudocylindriformis*. Although their aedeagi characteristics are not distinctive, the body size of the proposed two new species is different. Field observation revealed that niche utilization would be relevant for differentiating these closely related species.

## Introduction

The subfamily Cicindelinae of Carabidae consists of approximately 2,600 species (Pearson and Cassola 2005). Among them, *Cylindera* Westwood, 1831 is a diverse genus and widely distributed throughout the world. In Taiwan, including offshore islands such as Lanyu and Kingman, there are ten known *Cylindera* species and subspecies in four subgenera, including *C. cylindriformis* (Horn, 1912), *C. pseudocylindriformis* (Horn, 1913), *C. redunculata* Lin, 2017, and *C. sauteri* (Horn, 1912) in the subgenus Cylindera s. str.; *C. kaleea kaleea* (Bates, 1866), *C. kaleea angulimaculata* (Mandl, 1955), and *C. psilica psilica* (Bates, 1866) in the subgenus Ifasina; *C. elisae reductelineata* (Horn, 1912) and *C. elisae formosana* (Minowa, 1932) in the subgenus Eugrapha; and *C. shirakii* (Horn, 1927) in the subgenus Apterodela (Wiesner 1992; Werner et al. 2002; Löbl and Smetana 2003; Lin 2017). Additionally, however, *Apterodela* is either elevated to a full genus (Pearson et al. 2015; Puchkov and Matalin 2017) or is a subgenus within *Cylindera* based on a molecular phylogeny study (Gough et al. 2018). Some taxonomic issues of Taiwanese *Cylindera* are open to debate. For instance, *C. elisae reductelineata*, which is endemic to Taiwan, was differentiated genetically from the widespread lineage composed of other *C. elisae* subspecies, including the endemic subspecies *C. elisae formosana* (Sota et al. 2011), which is worth discussing. Moreover, the *C. sauteri* described commonly is in fact different from its type specimen (Werner et al. 2002).

*Cicindela sauteri* and *C. cylindriformis* were described by Horn (1912), and then *Prothyma pseudocylindriformis* was also described by Horn (1913). Schilder (1953) transferred *C. sauteri* and *C. cylindriformis* to the subgenus Jansenia and *Thopeutica* in genus *Cylindera*, respectively. In 1961, Rivalier classified *Cylindera* as nine subgenera and transferred *C. sauteri* and *C. cylindriformis* to subgenus Cylindera s. str. with the aedeagus illustration of *C. sauteri*. Referring to Rivalier’s opinion, Cassola (2002) transferred *P. pseudocylindriformis* to *Cylindera* s. str. based on the male genitalia characteristics. *Cylindera pseudocylindriformis* had been recorded in Vietnam (Horn 1929; Wiesner 1992; Cassola 2004), whereas Werner et al. (2002) considered it is endemic to Taiwan, and Wiesner et al. (2017) excluded it from the Cicindelinae checklist of Vietnam. Furthermore, one endemic new species, *C. redunculata* Lin, 2017, was described based on the elytral maculations compared with other *Cylindera* s. str. and *C. kaleea* (Lin 2017).

Recently, some specimens examined exhibit morphologically recognizable variations, which represents the possibility of undescribed *Cylindera* species in Taiwan. Field observation showed that *C. pseudocylindriformis*, inhabiting the soil slopes with gravels and litters near the forest, has a dark brownish body color and is seldom found on open ground. Several tiger beetles, however, collected from Pintung county, in southern Taiwan, are morphologically similar to *C. pseudocylindriformis* in elytral maculation pattern but have more obvious spots and lighter metallic coloration and inhabit the open forest trails. For *C. sauteri*, two forms were discovered in the specimens deposited in Museums für Naturkunde Berlin (MFNB): One is the commonly described *C. sauteri* with three spots on each elytron, and the other was collected in Kosempo, southern Taiwan, has a smaller body size and only two visible spots on each elytron, which are incongruent with the original description of *C. sauteri* by Horn (1912). Here, the ‘*sauteri*’ group inclusive of *C. sauteri* and Kosempo form was defined, and the ‘*pseudocylindriformis*’ group was considered to include *C. pseudocylindriformis* and the Pintung form. This study will test whether Kosempo and Pintung forms are undescribed species.

Tiger beetles were determined and described mainly based on morphological characters (Duran et al. 2018), especially labral shape, labral setae, elytral maculation, and male genitalia (Pearson and Vogler 2001; Pearson et al. 2015). Rivalier (1961) described the subgenus Cylindera s. str. as the following: (1) body slender; (2) maculation reduced and with longitudinal tendency when existing; (3) elytra usually with punctures; (4) underside hairs sparse; (5) proepisternum hairless; (6) labrum with 6–8 setae on margin; and (7) several species flightless due to reduced hind wings. However, the varied elytral maculation and coloration of tiger beetles might misjudge species identification and classification (Kaulbars and Freitag 1993; Cardoso and Vogler 2005; Woodcock et al. 2007), and lack of morphologically distinguishable characters might also overlook the existence of cryptic species (López-López et al. 2012, 2016; Duran et al. 2018).

Molecular evidence has been helpful for systematic work in tiger beetles, such as the sequences of cytochrome oxidase I (COI), 16S rDNA, and 28S rDNA (Cardoso et al. 2003; Sota et al. 2011; López-López et al. 2012, 2013, 2015, 2016; Jaskuła et al. 2016). The barcoding fragment of COI has been commonly used for species identification and delimitation (Hebert et al. 2003a, 2003b, 2004a). In the present study, more samples of Taiwanese *Cylindera* were acquired to examine the morphological characteristics, including genital characteristics, and to analyze the sequences of the two mitochondrial genes COI and 16S rDNA and one nuclear 28S rDNA. Based on molecular and morphological evidence, two new species of the aforementioned Kosempo and Pintung forms are thereby documented and described.

## Materials and methods

### Sampling

**Figure 1.**
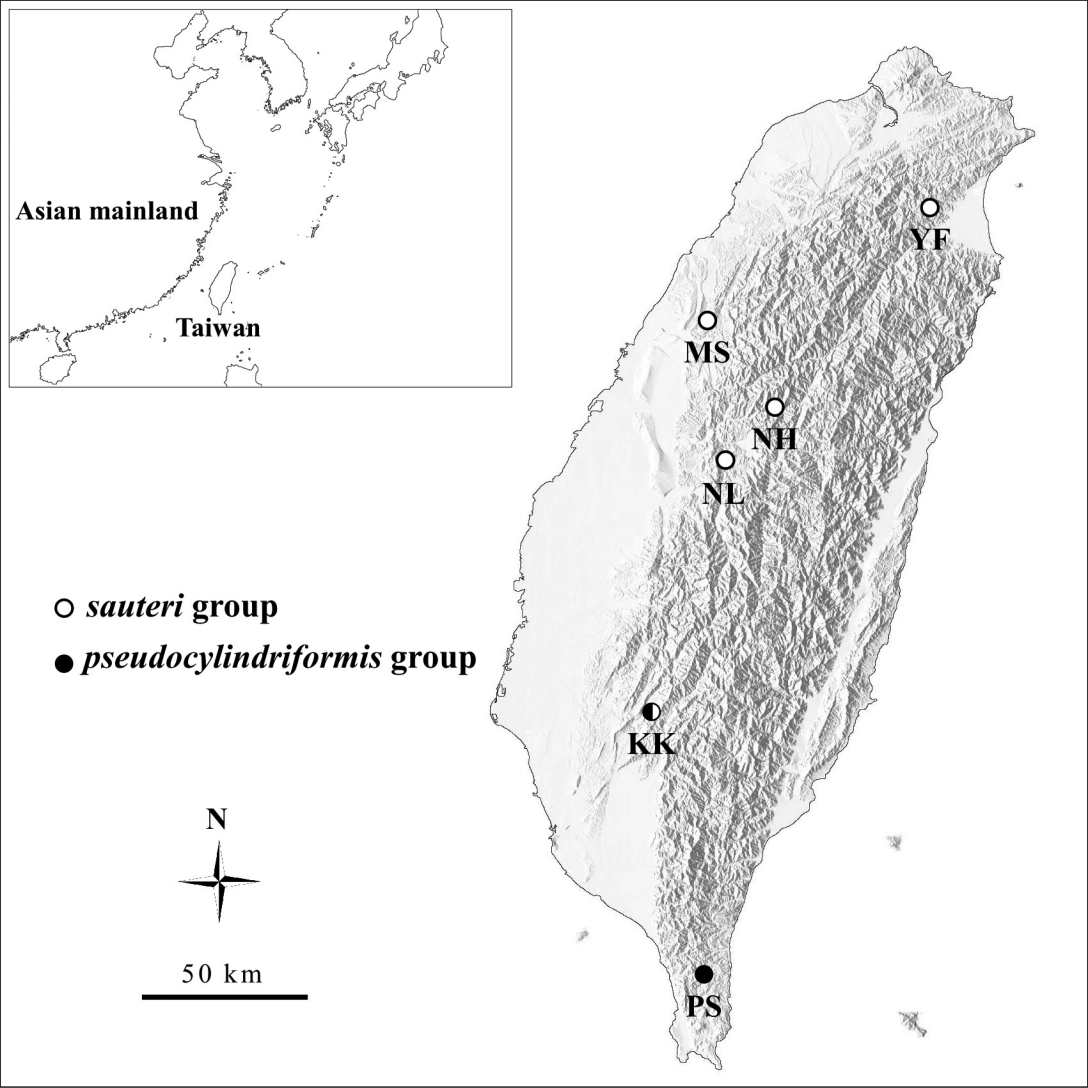
Sampling localities of the ‘*sauteri*’ and ‘*pseudocylindriformis*’ groups. Map was modified from the base map in website of Graduate Institute of Applied Geology of National Central University (http://gis.geo.ncu.edu.tw/earth/shade/twshades.htm).

*Cylindera* adults were collected by net around Taiwan. For the ‘*sauteri*’ group, 23 individuals of *C. sauteri* were sampled, and seven individuals of Kosempo form were collected in Jiaxian (Kosempo), Kaohsiung. As for the ‘*pseudocylindriformis*’ group, 11 individuals each of *C. pseudocylindriformis* and the Pintung form were collected. The sampling localities are shown in Fig. 1. Samples were preserved in 95% alcohol at -20 °C for morphology and DNA analysis. Some of them were processed as dry specimens for imaging after DNA extraction.

### Abbreviations

**NMNS** National Museum of Natural Science, Taichung, Taiwan

**NCHU** Department of Entomology of National Chung Hsing University, Taichung, Taiwan

**SDEI** Senckenberg Deutsches Entomologisches Institut, Müncheberg, Germany

**MFNB** Museum für Naturkunde, Berlin, Germany

**KK** Kosempo (Jiaxian), Kaohsiung, Taiwan

**KD** Daliao, Kaohsiung, Taiwan

**MS** Sanyi, Miaoli, Taiwan

**NH** Huisun Forest Area, Nantou, Taiwan

**NL** Lianhuachi, Nantou, Taiwan

**PS** Shuangliu Forest Recreation Area, Pintung, Taiwan

**YF** Fushan, Yilan, Taiwan

### DNA sequencing

Genomic DNA was extracted from the adult’s thoracic or leg muscle. A piece of tissue was ground in 50-μL solution of the QuickExtract DNA extraction kit (Epicentre Biotechnologies, Madison, WI), and then the sample solution was incubated at 65 °C for 10 min, followed by 98 °C for 2 min. After incubation, the sample solution was stored at -20 °C for polymerase chain reaction (PCR).

Primer pairs used to amplify COI, 16S rDNA, and 28S rDNA are listed in Table 1. PCR assay was performed in a 25-μL volume under the following conditions: first denaturation at 94 °C for 2 min, followed by 35 cycles of denaturation at 94 °C for 20 s, annealing at 45 °C or 50 °C for 40 s, and extension at 72 °C for 45 s. The final extension was at 72 °C for 10 min. The PCR products were purified by shrimp alkaline phosphatase/exonuclease I (USB Products, Affymetrix) treatment and then sequenced from both ends (COI) or single end (16S rDNA and 28S rDNA) by thermocycle sequencing using the BigDye terminator 3.1 sequencing kit (Applied Biosystems) following analyzed on an ABI 3730XL DNA Analyzer (Applied Biosystems). All sequence data were deposited in GenBank. Accession numbers for COI, 16S rDNA, and 28S rDNA are LC476849–LC476891, LC476978–LC477022, and LC477023–LC477066, respectively. Following Chakrabarty et al. (2013), the information on GenSeq and ranking of both ‘*sauteri*’ and ‘*pseudocylindriformis*’ groups are listed in Suppl. material 1: Table S1.

**Table 1.** The primer pairs used in PCR.

Genes	Primers	Sequences (5’–3’)	References
COI	Col46 (+)	AACCATAAAGATATTGGAAC	Tsai et al. 2014
	Col731 (-)	CCAAAAAATCAAAATAAATGTTG	Tsai et al. 2014
	LCO1490 (+)	GGTCAACAAATCATAAAGATATTGG	Folmer et al. 1994
	HCO2198 (-)	TAAACTTCAGGGTGACCAAAAAATCA	Folmer et al. 1994
16S rDNA	16SR21(+)	GCCTGTTTATCAAAAACAT	Yeh et al. 2004
	16S22 (-)	CCGGTCTGAACTCAGATCA	Yeh et al. 2004
28S rDNA	28Se (+)	TCCGTAACTTCGGAACAAGGATT	Lin et al. 2003
	28Sf (-)	TGTACCGCCCCAGTCAAACT	Lin et al. 2003

### Phylogenetic inference

DNA sequences were aligned using the ClustalW multiple alignment program and then edited in Bioedit 7.0 (Hall 1999). The pairwise genetic distances of three genes within both groups were calculated using Kimura 2-parameter model in MEGA 7.0 (Kumar et al. 2016). Pairwise distances of COI were used to determine the barcoding gap between forms, which is helpful to delimit different species (Hebert et al. 2004b). In addition, the maximum intra-taxa COI sequence divergence and minimum inter-taxa COI sequence divergence were also applied (Meier et al. 2008).

*Cylindera redunculata* belonging to the same subgenus Cylindera s. str. was used as the phylogenetic outgroup. Sequences of COI, 16S rDNA, and 28S rDNA were used to perform phylogenetic analyses. The best-fit substitution models applied to different genes were inferred in jModelTest 2.1 (Darriba et al. 2012) using the Bayesian information criterion (BIC). The best-fit models for COI, 16S DNA, and 28S rDNA were TPM2uf+I, TPM1uf, and F81 for the sauteri group and HKY+I, HKY, and F81 for the pseudocylindriformis group, respectively. Bayesian inference (BI) was conducted using MrBayes 3.2.6 (Ronquist et al. 2012). The partitioned analyses of the combined data (COI+16S rDNA+28S rDNA) were set up. Markov chain Monte Carlo (MCMC) methods were conducted for 1×10^6^ generations, sampling every 1000 generations; then, the analyses were settled when the average standard deviation of split frequencies < 0.01. The 25% trees were burn-in to obtain a consensus tree. The maximum likelihood (ML) analyses were performed on an online version of PhyML 3.0 (http://www.atgc-montpellier.fr/phyml/) (Guindon et al. 2010) with 1000 bootstrap replications, and the best-fit models were searched using BIC by Smart Model Selection (Lefort et al. 2017).

Divergence time estimation was performed in BEAST 2.5.1 (Bouckaert et al. 2018) using the combined data of COI, 16S rDNA, and 28S rDNA. The substitution models for partition were the same as BIs. Calibration rates of COI, 16S rDNA, and 28S rDNA were 3.34%, 0.76% (Pons et al. 2011), and 0.17% (Sota et al. 2011) per lineage per million years, respectively; and strict clock was applied. Parameters of the prior panel were set as the default. MCMC chain length was 1×10^8^ generations sampling every 1000 steps. The output results were assessed in Tracer 1.6 to examine the effective sample sizes as optimal, i.e., > 200, or not. The tree files were combined in LogCombiner 2.5.2 with the removal of 10% burnin, and then TreeAnnotator 2.5.1 was used to generate a maximum credibility tree with median node heights.

### Morphology analyses

Body lengths were measured using Microsight 4.1.2 connected with a Canon EOS 800D camera (Tokyo, Japan); this equipment was also used for imaging aedeagi. Specimens images were taken using a Nikon Coolpix B700 camera (Tokyo, Japan) with a Raynox DCR-250 macrolens (Tokyo, Japan). To avoid influencing the measurement by head pose, lengths of the pronotum and elytron were applied as body length. R 3.4.3 (R Core Team 2017) was used to conduct two-sample Wilcoxon rank-sum tests to test whether the body lengths of the same sex between different forms of the two species group were different statistically. A two-tailed t-test and *p* ≤ 0.05 was considered significant.

Male genitalia of both forms were dissected and dipped in 10% KOH solution at room temperature for 12 h. The treated genitalia were preserved in glycerol for imaging and then described (Shi et al. 2013). The terminology of genital structures followed Freitag et al. (1985) and Acciavatti and Pearson (1989).

## Results

### Phylogenetic inferences

**Figure 2.**
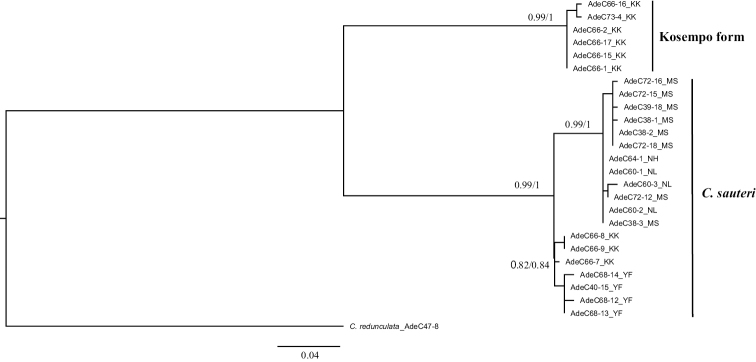
ML tree of the sauteri group reconstructed based on the combined data of COI, 16S rDNA, and 28S rDNA with ML bootstrap values (left) and BI posterior probability (right) that are shown when > 0.5.

**Figures 3, 4.**
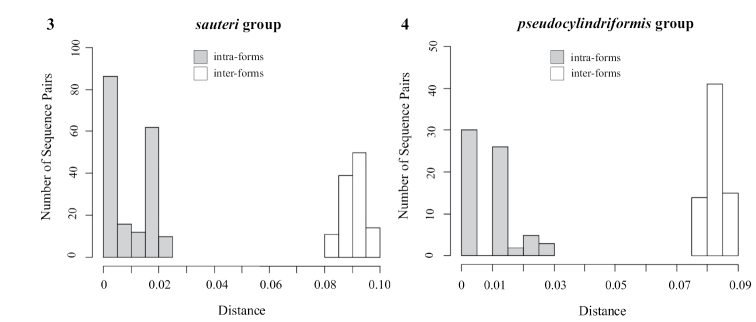
Frequency distributions of COI pairwise distance of the sauteri group (**3**) and the pseudocylindriformis group (**4**), showing the barcoding gaps.

**‘*sauteri*’ group.** Twenty-five sequences of COI, 16S rDNA, and 28S rDNA with a length of 660 bp, 472–473 bp, and 850 bp, respectively, were obtained and aligned. The combined data indicated that Kosempo form and *C. sauteri* were reciprocally monophyletic groups with high support values (ML = 0.99, BI = 1 for each of them) (Fig. 2). ML trees of COI, 16S rDNA, and 28S rDNA are shown in Suppl. material 2: Figs S1, S2, and S3, respectively, and their topology resolutions show the reciprocal monophyly of Kosempo form and *C. sauteri*. These forms diverged approximately 1.36 million years ago (Mya) (Suppl. material 2: Fig. S4). The minimum COI distance between them was 0.083, and the maximum intra-form distance was 0.023 (Suppl. material 1: Table S2). The barcoding gap existed clearly (Fig. 3). Pairwise distances of 16S rDNA and 28S rDNA are shown in Suppl. material 1: Tables S3 and S4, respectively.

**Figure 5.**
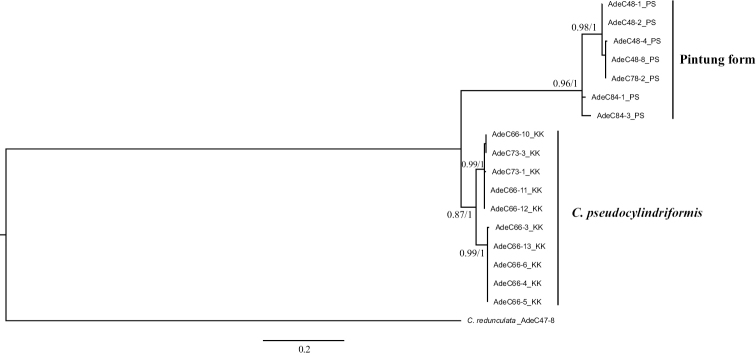
ML tree of the pseudocylindriformis group reconstructed based on the combined data of COI, 16S rDNA, and 28S rDNA with ML bootstrap values (left) and BI posterior probability (right) that are shown when > 0.5.

**‘*pseudocylindriformis*’ group.** There were 17, 19, and 18 sequences of COI, 16S rDNA, and 28S rDNA of lengths 661 bp, 471 bp, and 848 bp, respectively, that were obtained and aligned. The ML tree based on combined data showed the reciprocal monophyly of Pintung form and *C. pseudocylindriformis* with high support of values (ML = 0.96, BI = 1 for Pintung form; ML = 0.87, BI = 1 for *C. pseudocylindriformis*) (Fig. 5). Both ML trees of COI (Suppl. material 2: Fig. S5) and 16S rDNA (Suppl. material 2: Fig. S6) also showed that these forms were reciprocally monophyletic. However, the phylogenetic resolution inferred from 28S rDNA showed Pintung form monophyly only (Suppl. material 2: Fig. S7). Molecular dating placed the differentiation event between the two at approximately 1.26 Mya (Suppl. material 2: Fig. S8). The minimum inter-form and maximum intra-form distances of COI were 0.076 and 0.028, respectively (Suppl. material 1: Table S5), indicating existence of the barcoding gap (Fig. 4). Pairwise distances of 16S rDNA and 28S rDNA are shown in Suppl. material 1: Tables S6 and S7, respectively.

### Morphology

**Figure 6.**
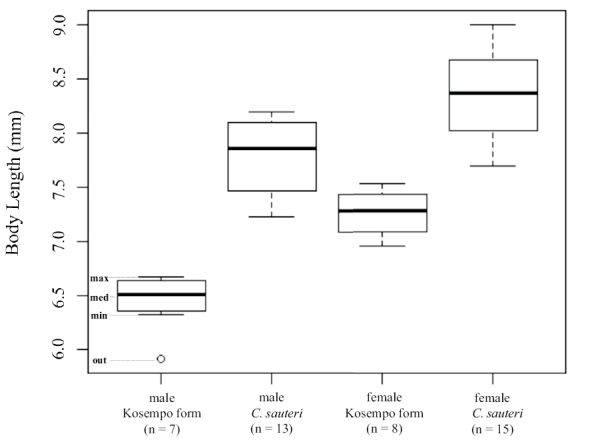
Body length (pronotum and elytron) of the sauteri group. Legends: **max** maximum; **med** median; **min** minimum; **out** outlier.

Morphological and genital characteristics described for sauteri and pseudocylindriformis groups were as follows:

**‘*sauteri*’ group.** Body lengths (pronotum and elytron) of Kosempo form were 5.91–6.67 mm (mean = 6.44 mm, n = 7) for males and 6.95–7.53 mm (mean = 7.26 mm, n = 8) for females, and the lengths of *C. sauteri*, including the specimens borrowed from MFNB and our collections, were 7.23–8.19 mm (mean = 7.79 mm, n = 13) for males and 7.69–9.00 mm (mean = 8.35 mm, n = 15) for females (Fig. 6). In both sexes, body lengths of *C. sauteri* were significantly larger than those of Kosempo form (*p* = 0.0004 for males; *p* = 0.000004 for females).

**Figures 7, 8.**
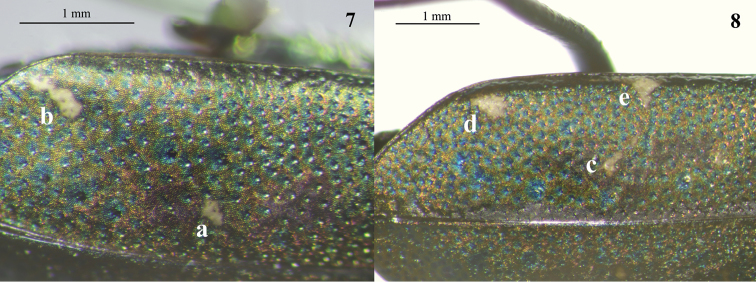
The elytral maculations (left elytron). **7** Kosempo form lacks any spot on the middle elytral edge and has one spot near suture (**a**) and one subapical spot (**b**) **8***Cylindera sauteri* has one spot near suture (**c**), one subapical spot (**d**), and one triangular spot on the elytral middle edge (**e**).

Elytral maculation of Kosempo form mostly included two spots on each elytron: One spot near elytral suture (Fig. 7, a), and one subapical spot at subapical corner of elytron (Fig. 7, b). However, one of 15 individuals of Kosempo form possessed visible posthumeral spots. *Cylindera sauteri* possessing three spots on each elytron: one spot near suture (Fig. 8, c), one subapical spot at subapical corner (Fig. 8, d), and one spot at middle edge of elytron (Fig. 8, e). Spot near suture and spot at middle edge usually connected very weakly. Posthumeral spot absent or hardly visible in all 23 specimens of *C. sauteri*.

**Figures 9, 10.**
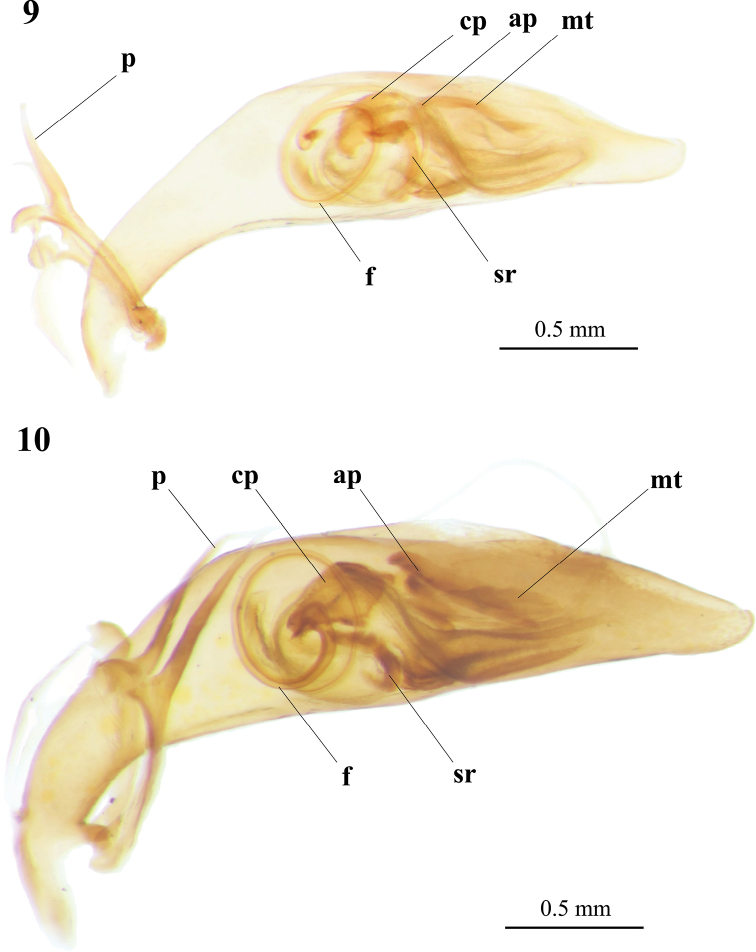
Digital image of aedeagus in left view of Kosempo form (AdeC66-1) (**9**) and *Cylindera sauteri* (**10**). Abbreviations: **ap** arciform piece; **cp** central plate; **f** flagellum; **p** paramere; **mt** medial tooth; **sr** stiffening rib.

Male genitalia were very similar in external shape and inner sac between Kosempo form (n = 3) and *C. sauteri* (n = 8) but different in size (Figs 9, 10). Basal portion of aedeagus short and slightly bent, median portion widened, apical portion narrow gradually, apical top rounded. Paramere (p) slender, acanthoid. On the left view of aedeagus, base of flagellum (f) convoluted spirally; stiffening rib (sr) near base of flagellum with two upcurved ends; central plate (cp) irregular; medial tooth (mt) and arciform piece (ap) oblique near subapical apex and overlapping.

**Figure 11.**
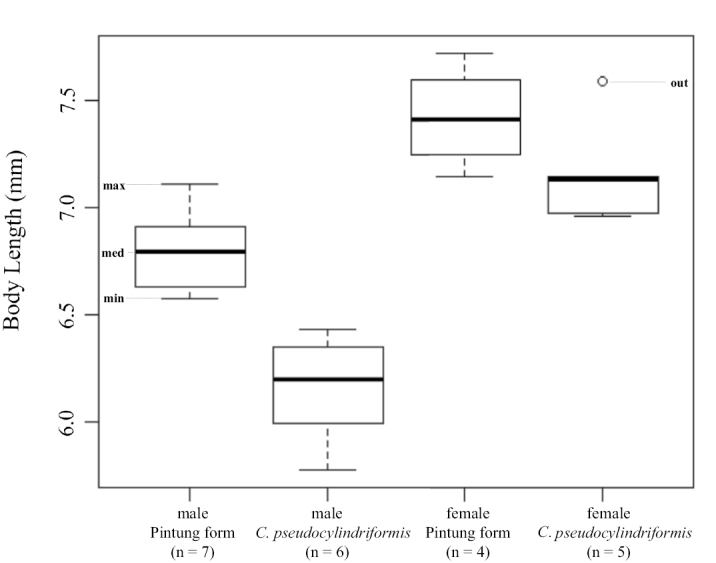
Body length (pronotum and elytron) of the pseudocylindriformis group. Abbreviations: **max** maximum; **med** median; **min** minimum; **out** outlier.

**‘*pseudocylindriformis*’ group**. Body lengths (pronotum and elytron) of Pintung form were 6.57–7.11 mm (mean = 6.79 mm, n = 7) for males and 7.14–7.72 mm (mean = 7.42 mm, n = 4) for females and of *C. pseudocylindriformis*, 5.77–6.43 mm (mean = 6.16 mm, n = 6) for males and 6.96–7.59 mm (mean = 7.16 mm, n = 5) for females (Fig. 11). Pintung form was significantly larger than *C. pseudocylindriformis* in males (*p* = 0.001166) but not in females (*p* = 0.14).

The pattern of elytral maculation of Pintung form and *C. pseudocylindriformis* almost identical (details provided below Figs 26–28). Humeral spot and posthumeral spot visible in both forms. Spot at middle edge connected to spot near suture very weakly but connected together in one *C. pseudocylindriformis* and three Pintung form specimens. Apical lunula visible, and its subapical portion thickened in both forms, but apical end near suture thickened only in Pintung form.

**Figures 12, 13.**
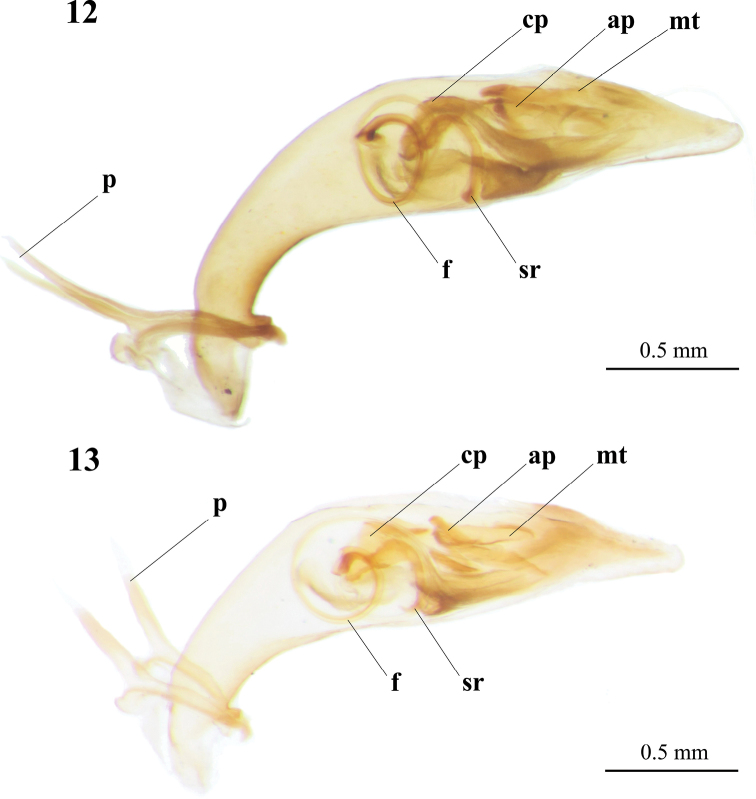
Digital image of aedeagus in left view of Pintung form (**12**) and *Cylindera pseudocylindriformis* (**13**). Abbreviations: **ap** arciform piece; **cp** central plate; **f** flagellum; **p** paramere; **mt** medial tooth; **sr** stiffening rib.

Male genitalia similar in morphology between *C. pseudocylindriformis* (n = 5) and Pintung form (n = 4) and even similar to sauteri group. External shape slender, median portion widened, apical portion narrow gradually with a rounded apical top, basal portion slightly shorter in *C. pseudocylindriformis* and slenderer in Pintung form. Paramere (p) slender, acanthoid. Structures of inner sac almost identical in both forms, base of flagellum (f) convoluted spirally on left view; stiffening rib (sr) near base of flagellum; central plate (cp) irregular; medial tooth (mt) and arciform piece (ap) oblique near subapical apex and overlap partially (Figs 12, 13).

## Discussion

Phylogenetic trees inferred from molecular combined data show that both forms in sauteri and pseudocylindriformis groups are monophyletic reciprocally with high support values (Figs 2, 5). The weak phylogenetic resolution inferred from the 28S rDNA fragments of Pintung form and *C. pseudocylindriformis* might be due to the conserved property of 28S rDNA applying to resolve the relationship of closely related species (Guerra et al. 2016; Tsai and Yeh 2016). Phylogenetic inferences, molecular dating, and the deep barcoding gap indicate that the different forms are genetically distinct.

According to the original description of *C. sauteri* (Horn 1912), its elytron is garnished with two or three testaceous maculae: one is discoidal and very tiny near the middle suture, another triangular one is approximately at the middle edge (sometimes deficient), and the third is oblique on the subapical corner. This is the main difference from the Kosempo form, which lacks the triangular spot at the middle edge of elytron and has an elongated subapical spot (Fig. 7b). The triangular spot and rounded subapical spot of *C. sauteri* were illustrated in the line drawing by Horn (1938). Some *C. sauteri* individuals have a very tiny or obscure triangular macula, but this spot does not disappear completely. As for pseudocylindriformis group, the elytral apical lunula of Pintung form is thickened in the apical portion near the elytral suture, but it stays linear and slender in *C. pseudocylindriformis*. The line drawing of syntype of *C. pseudocylindriformis* also shows this character of apical lunula (Cassola 2002). Moreover, Pintung form is more metallic brown than *C. pseudocylindriformis*. Their aedeagi are poorly distinctive (Figs 12, 13); however, male genitalia of *C. sauteri* and *C. pseudocylindriformis* are also nearly identical (Rivalier 1961; Cassola 2002), indicating morphologic conservation of male genitalia among closely related *Cylindera* species.

Based on the genetic distinction and stable morphological differences, Kosempo form and Pintung form could be recognized as two undescribed species. In the present study, Kosempo form of the sauteri group is named *Cylindera ooa* sp. nov., and Pintung form of the pseudocylindriformis group is named *Cylindera autumnalis* sp. nov. Moreover, *C. ooa* sp. nov. seems to be confined to the Jiaxian region, but *C. sauteri* is widely distributed across the Taiwan Island. The type localities of *C. sauteri* are Kosempo (Jiaxian, Kaohsiung) and Taihorin (Dalin, Chiayi) (Horn 1912). Unfortunately, we could not examine the type specimens of *C. sauteri* because they were on loan till the time of writing this manuscript. It is necessary to clarify whether the type series of *C. sauteri* include *C. ooa* sp. nov. specimens. Even so, the recognizable morphological characters proposed in this study will be helpful in distinguishing them.

Ecological niche differentiation in sympatric closely related species could be related to morphological divergence such as body size because of different resource utilization (Wilson 1975; Pearson and Stemberger 1980; Dangalle et al. 2013). *Cylindera autumnalis* sp. nov. inhabiting open forest trails might not overlap with *C. pseudocylindriformis* preferring soil slopes with more cover. However, *C. ooa* sp. nov. and *C. sauteri* occupy similar habitat types of soil slopes with some gravel and little vegetation, and both can be found in Jiaxian area in the same season although a field survey did not observe the sympatric distribution of *C. sauteri* and *Cyl ooa* sp. nov. nor that of *C. autumnalis* sp. nov. and *C. pseudocylindriformis*. Notably, the body size is significantly different in both proposed new species from their closely related species. The body size would be one of the characters shaped by the process of niche differentiation and speciation. In addition, physiological differences (Schultz and Hadley 1987), oviposition behaviors (Hoback et al. 2000, 2001), and thermoregulatory behaviors (Brosius and Higley 2013) are also relevant to niche differentiation of tiger beetles.

Moreover, the subgenus Cylindera s. str. of Taiwan possessing a comparatively longitudinally elongated labrum, thoracic proepisternum with hairs (*C. sauteri* and *C. ooa* sp. nov.), well developed hind wings for flight, and a more slender body seems morphologically distinct from the other members of the subgenus Cylindera s. str. Gough et al. (2018) showed the subgenus Cylindera s. str. was polyphyletic because the subgenus Cylindera s. str. of Palearctic and Oriental was a sister to the subgenus Ifasina, whereas its Nearctic fauna was nested with other genera.

## Taxonomy

### Cylindera (Cylindera) ooa sp. nov.

Taxon classificationAnimaliaColeopteraCarabidae

DF115E9B312F57BCB762D29A3047717C

http://zoobank.org/7D37BBD1-3BDA-4C13-9F2C-5C47412A21D9

#### Type material.

**Figures 14–16.**
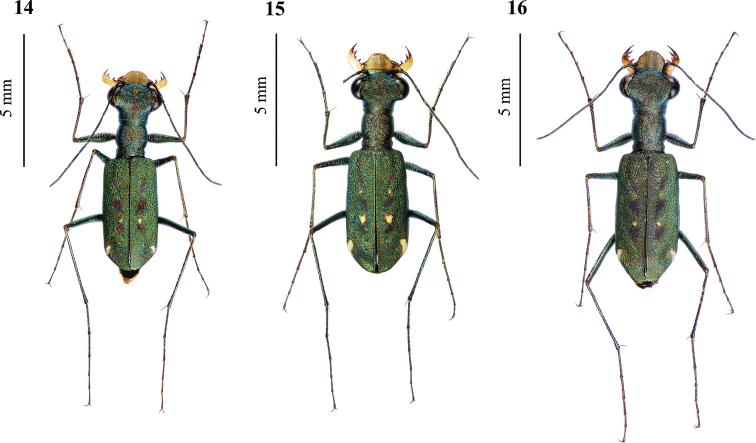
Type specimens of *Cylindera ooa* sp. nov. **14** male holotype (AdeC66-1) **15** male paratype (AdeC66-15) exhibiting visible posthumeral spots **16** female paratype (AdeC66-2).

***Holotype:*** male (Fig. 14; specimen code: AdeC66-1; dry pinned, with aedeagus in glycerol in a separated microvial labeled “AdeC66-1”): Taiwan, Kaohsiung, Jiaxian, Liuyi Mountain, altitude 400–500 m, 17 May 2018, Ming-Hsun Chou leg. Original label: “Locality: 高雄甲仙六義山 / Date: 2018.V.17 / Collector: 周明勳 / Code: AdeC66-1”; “NCHU 0011-0735”. Dry specimen and aedeagus of holotype deposited in NCHU. ***Paratypes***: 1 male (Fig. 15; specimen code: AdeC66-15; dry pinned, with aedeagus in glycerol in a separated microvial) and 3 females (specimen code: AdeC66-2 (Fig. 16), AdeC66-16, and AdeC66-17, respectively; dry pinned, with genitalia in glycerol in a separated microvial, respectively): same collecting information as for holotype. 1 male (specimen code: AdeC73-4; dry pinned, with aedeagus in glycerol in a separated microvial): Taiwan, Kaohsiung, Jiaxian, Liuyi Mountain, altitude 400–500 m, 23 Jun. 2018, Ming-Hsun Chou leg. Above dry specimens and genitalia of paratypes deposited in NMNS. 3 females (dry pinned, labeled “Paratype-MFNB-01”, “Paratype-MFNB-02”, and “Paratype-MFNB-07”, respectively): Taiwan, Kaohsiung, Jiaxian, 9–17 May 1908, Sauter S.V. leg. 1 male (dry pinned, labeled “Paratype-MFNB-03”): Taiwan, Kaohsiung, Jiaxian, 17–23 May 1908, Sauter S.V. leg. 1 male (dry pinned, labeled “Paratype-MFNB-04”): Taiwan, Kaohsiung, Jiaxian, 2–14 May 1908, Sauter S.V. leg. 1 male (dry pinned, labeled “Paratype-MFNB-05”): Taiwan, Kaohsiung, Jiaxian, 1–5 May 1908, Sauter S.V. leg. 1 female (dry pinned, labeled “Paratype-MFNB-06”): Taiwan, Kaohsiung, Jiaxian, 1–5 May 1908, Sauter S.V. leg. Above dry specimens of paratypes deposited in MFNB. Original labels of paratypes see Table 2.

**Table 2.** Original labels of type materials.

Species	Code	Type of type	Original label	In English
*Cylindera ooa*	AdeC66-1	Holotype	Locality: 高雄甲仙六義山	Locality: Kaohsiung, Jiaxian, Liuyi Mountain
			Date: 2018.V.17	Date: 2018.V.17
			Collector: 周明勳	Collector: Ming-Hsun Chou
			Code: AdeC66-1	Code: AdeC66-1
	AdeC66-2	Paratype	Locality: 高雄甲仙六義山	Locality: Kaohsiung, Jiaxian, Liuyi Mountain
			Date: 2018.V.17	Date: 2018.V.17
			Collector: 周明勳	Collector: Ming-Hsun Chou
			Code: AdeC66-2	Code: AdeC66-2
	AdeC66-15	Paratype	Locality: 高雄甲仙六義山	Locality: Kaohsiung, Jiaxian, Liuyi Mountain
			Date: 2018.V.18	Date: 2018.V.18
			Collector: 周明勳	Collector: Ming-Hsun Chou
			Code: AdeC66-15	Code: AdeC66-15
	AdeC66-16	Paratype	Locality: 高雄甲仙六義山	Locality: Kaohsiung, Jiaxian, Liuyi Mountain
			Date: 2018.V.18	Date: 2018.V.18
			Collector: 周明勳	Collector: Ming-Hsun Chou
			Code: AdeC66-16	Code: AdeC66-16
	AdeC66-17	Paratype	Locality: 高雄甲仙六義山	Locality: Kaohsiung, Jiaxian, Liuyi Mountain
			Date: 2018.V.18	Date: 2018.V.18
			Collector: 周明勳	Collector: Ming-Hsun Chou
			Code: AdeC66-17	Code: AdeC66-17
	AdeC73-4	Paratype	Locality: 高雄甲仙六義山	Locality: Kaohsiung, Jiaxian, Liuyi Mountain
			Date: 2018.VI.23	Date: 2018.VI.23
			Collector: 周明勳	Collector: Ming-Hsun Chou
			Code: AdeC73-4	Code: AdeC73-4
	Paratype-MFNB-01	Paratype	“Formosa / Kosempo / Sauter S.V. / 9.–17. V. 08”	
			“Zool. Mus. Berlin”	
	Paratype-MFNB-02	Paratype	“Formosa / Kosempo / Sauter S.V. / 9.–17. V. 08”	
			Zool. Mus. Berlin	
	Paratype-MFNB-03	Paratype	“Formosa / Kosempo / Sauter S.V.”	
			“17.–23. V. 08”	
			“Zool. Mus. Berlin”	
	Paratype-MFNB-04	Paratype	“Formosa / Kosempo / Sauter S.V.”	
			“2.–14. VI. 08”	
			“Zool. Mus. Berlin”	
	Paratype-MFNB-05	Paratype	“Formosa / Kosempo / Sauter S.V.”	
			“1.–5. V. 08”	
			“Zool. Mus. Berlin”	
	Paratype-MFNB-06	Paratype	“Formosa / Kosempo / Sauter S.V.”	
			“1.–5. V. 08”	
			“Zool. Mus. Berlin”	
	Paratype-MFNB-07	Paratype	“Formosa / Kosempo / Sauter S.V. / 9.–17. V. 08”	
			“Zool. Mus. Berlin”	
*Cylindera autumnalis*	AdeC48-1	Paratype	Locality: 屏東雙流森林遊樂區	Locality: Pintung, Shuangliu Forest Recreation Area
			Date: 2017.VIII.10	Date: 2017.VIII.10
			Collector: 周明勳	Collector: Ming-Hsun Chou
			Code: AdeC48-1	Code: AdeC48-1
	AdeC48-2	Paratype	Locality: 屏東雙流森林遊樂區	Locality: Pintung, Shuangliu Forest Recreation Area
			Date: 2017.VIII.10	Date: 2017.VIII.10
			Collector: 周明勳	Collector: Ming-Hsun Chou
			Code: AdeC48-2	Code: AdeC48-2
	AdeC48-4	Paratype	Locality: 屏東雙流森林遊樂區	Locality: Pintung, Shuangliu Forest Recreation Area
			Date: 2017.VIII.10	Date: 2017.VIII.10
			Collector: 周明勳	Collector: Ming-Hsun Chou
			Code: AdeC48-4	Code: AdeC48-4
	AdeC48-5	Paratype	Locality: 屏東雙流森林遊樂區	Locality: Pintung, Shuangliu Forest Recreation Area
			Date: 2017.VIII.10	Date: 2017.VIII.10
			Collector: 周明勳	Collector: Ming-Hsun Chou
			Code: AdeC48-5	Code: AdeC48-5
	AdeC48-8	Paratype	Locality: 屏東雙流森林遊樂區	Locality: Pintung, Shuangliu Forest Recreation Area
			Date: 2017.VIII.11	Date: 2017.VIII.11
			Collector: 周明勳	Collector: Ming-Hsun Chou
			Code: AdeC48-8	Code: AdeC48-8
	AdeC78-1	Paratype	Locality: 屏東雙流森林遊樂區	Locality: Pintung, Shuangliu Forest Recreation Area
			Date: 2018.VII.21	Date: 2018.VII.21
			Collector: 周明勳	Collector: Ming-Hsun Chou
			Code: AdeC78-1	Code: AdeC78-1
	AdeC78-2	Paratype	Locality: 屏東雙流森林遊樂區	Locality: Pintung, Shuangliu Forest Recreation Area
			Date: 2018.VII.21	Date: 2018.VII.21
			Collector: 周明勳	Collector: Ming-Hsun Chou
			Code: AdeC78-2	Code: AdeC78-2
	AdeC84-1	Paratype	Locality: 屏東雙流森林遊樂區	Locality: Pintung, Shuangliu Forest Recreation Area
			Date: 2018.IX.03,	Date: 2018.IX.03,
			Collector: 周明勳	Collector: Ming-Hsun Chou
			Code: AdeC84-1	Code: AdeC84-1
	AdeC84-2	Paratype	Locality: 屏東雙流森林遊樂區	Locality: Pintung, Shuangliu Forest Recreation Area
			Date: 2018.IX.03,	Date: 2018.IX.03,
			Collector: 周明勳	Collector: Ming-Hsun Chou
			Code: AdeC84-2	Code: AdeC84-2
	AdeC84-3	Holotype	Locality: 屏東雙流森林遊樂區	Locality: Pintung, Shuangliu Forest Recreation Area
			Date: 2018.IX.03,	Date: 2018.IX.03,
			Collector: 周明勳	Collector: Ming-Hsun Chou
			Code: AdeC84-3	Code: AdeC84-3

#### Type locality.

Taiwan, Kaohsiung, Jiaxian, Liuyi Mountain.

#### Diagnosis.

**Figure 17.**
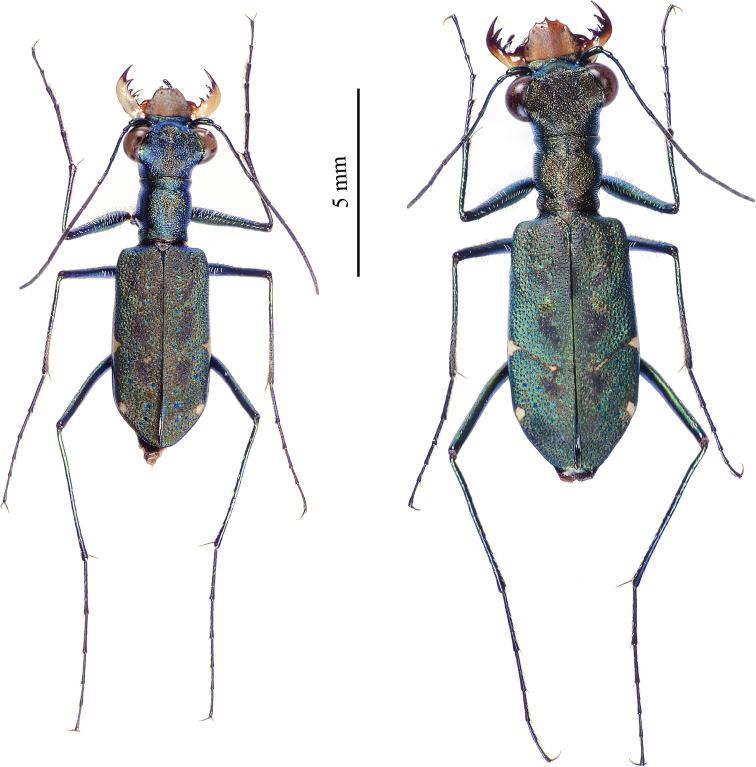
Dorsal habitus of *Cylindera sauteri* (left – male; right – female).

**Figures 18–21.**
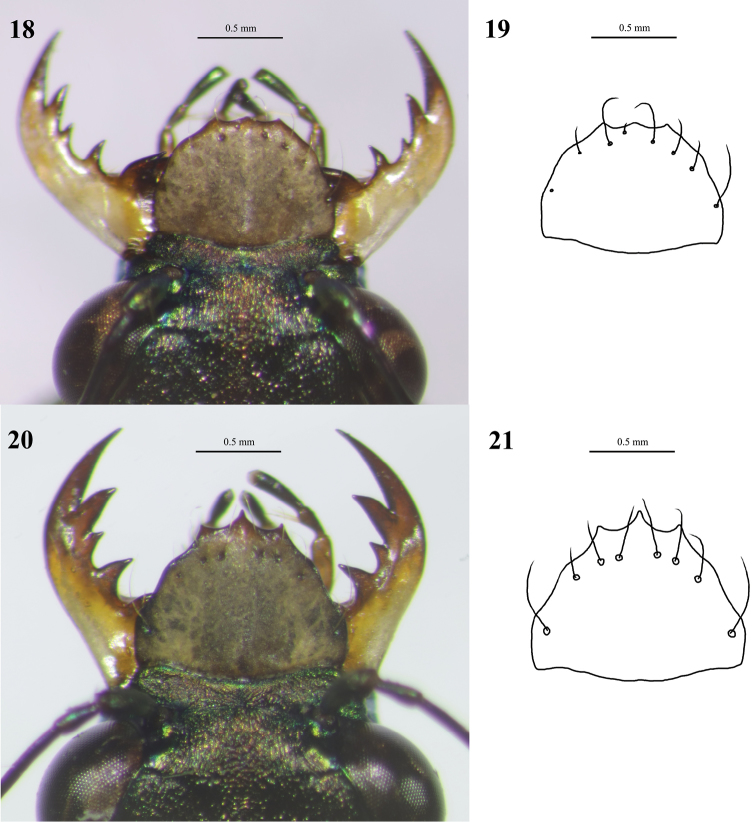
Digital images (left) and line drawings (right) of labra of *Cylindera ooa* sp. nov. **18, 19** male (holotype, AdeC66-1) **20, 21** female (paratype, AdeC66-2).

**Figures 22–25.**
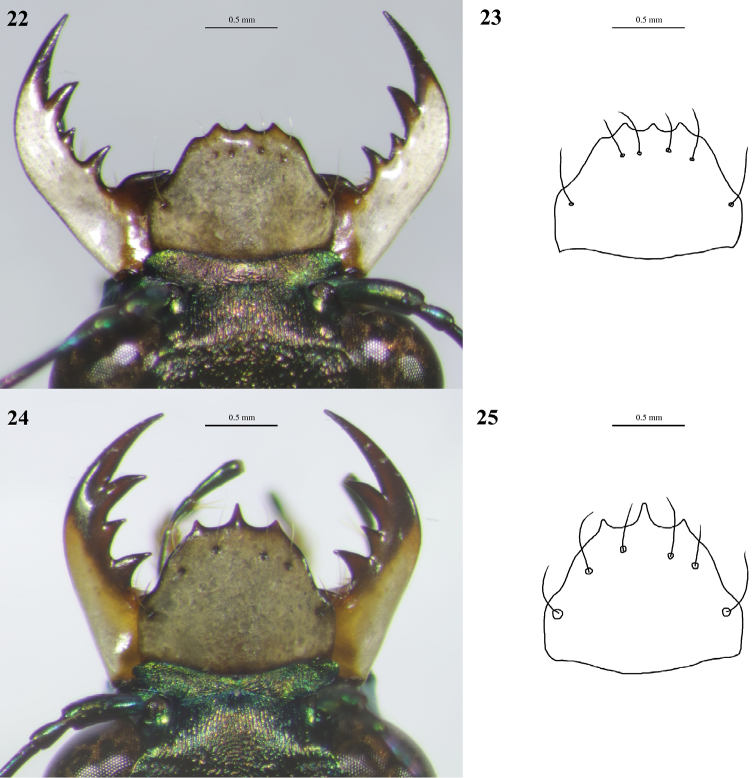
Digital images (left) and line drawings (right) of labra of *Cylindera sauteri***22, 23** male **24, 25** female.

*Cylindera ooa* sp. nov. can be recognized based on its elongated subapical spots and no any spot at the middle edges of elytra. This species is very similar to *C. sauteri* (Fig. 17) morphologically but can be distinguished from the latter by their elytral maculation, labrum, and body size. *Cylindera sauteri* has a nearly triangular spot at the middle margin of elytron, and its subapical spot is comparatively tiny or rounded. In contrast, the middle elytral margin of *C. ooa* sp. nov. does not have any spot, and its subapical spot is comparatively longer than that of *C. sauteri* (Figs 7, 8). The labrum of *C. ooa* sp. nov. is more straight laterally and has five or six preapical setae (Figs 18–21), but the labrum of *C. sauteri* is concave in lateral sides and has four or five preapical setae (Figs 22–25). Moreover, the body sizes of *C. sauteri*, as well as male genitalia, are usually larger than those of *C. ooa* sp. nov. (Figs 6, 9, 10).

#### Description.

***Head*** brownish patina with blue or green luster but more brownish when alive; vertex, frons, and genae almost glabrous except two setae on canthus and anterior portion of vertex, respectively; rugae longitudinal along frons, canthi, vertex, and lateral neck, and gradually becoming transverse on genae; frons and central vertex microsculptured; clypeus brownish patina and microsculptured. ***Compound eyes*** protruding and globular. ***Antennae*** long and filiform; scape with one apical seta; 1–4 antennomeres metallic bronze; 5–11 ones dark. ***Mandibles*** testaceous with dark teeth, exceeding labrum when closed. ***Maxillary palps*** dark testaceous with metallic luster, except last two palpomeres metallic dark green. ***Labial palps*** testaceous; last palpomere metallic dark green. ***Labrum*** testaceous; anterior portion narrow and tridentate; middle tooth longer than other two in female, shorter than or equivalent to others in male; margin with 5–6 preapical and two lateral setae (Figs 18–21). ***Pronotum*** cylindrical and brownish patina with blue or green luster but more brownish when alive; dorsum microsculptured and rugose transversely, with one transverse groove on each anterior and posterior portions connected with one longitudinal obscure groove; lateral sides little rounded. ***Elytra*** brownish patina but more brownish when alive, marked with many scattered punctures; three obscure brownish patches wiping longitudinally near suture; each elytron with usually two white or testaceous spots, one rounded or irregular near suture, one elongated a little and oblique on subapical corner; posthumeral spots usually absent or unobvious but visible in some individuals (Fig. 15). ***Legs*** long; trochanters brownish; coxae, femurs and tibias metallic greenish bronze; tarsi dark greenish with purple luster, pro-tarsi sexually dimorphic, basal 1–3 tarsomeres with dense brush-like ventral setae and wider than last two tarsomeres in male, all pro-tarsomeres equivalent in width roughly and without brush-like ventral setae in female; some white hairs on femurs and coxae, one long seta on pro-, mesocoxae, pro- and mesotrochanters. ***Thoracic proepisternum*** brownish patina with greenish luster, longitudinally rugose, with 2–4 hairs on lower portion. ***Prosternum*** brownish patina with greenish luster, transversally rugose, glabrous. ***Mesoepisternum*** brownish patina with greenish luster, longitudinally depressed and coarsely rugose, sometimes with rare hairs. ***Mesosternum*** brownish patina with greenish luster, transversally rugose, sometimes with rare hairs. ***Metepisternum*** brownish patina with greenish luster, coarsely sculptured, with a few hairs. ***Metasternum*** dark bronze with greenish luster, microsculptured, covered by many white hairs on both sides. ***Abdomen sternum*** dark green with metallic greenish reflection and with scattered tiny hairs. ***Aedeagus*** of holotype shown in Fig. 8. Description same as Results.

#### Etymology.

Jiaxian, the type locality, is famous for taro cultivation and products. The Taiwanese pronunciation of taro is ōo-á, so it was applied as specific name.

#### Distribution.

Only known from type locality.

#### Ecology.

Habitat of *C. ooa* sp. nov. is similar to *C. sauteri* that they inhabit soil slopes with some gravels and covered by a few vegetation in or near forest. *Cylindera sauteri* can also be found in Jiaxian, but we did not observe them in the same habitat. *Cylindera pseudocylindriformis* inhabits soil slopes as well and sometimes overlaps with *C. ooa* sp. nov.

### Cylindera (Cylindera) autumnalis sp. nov.

Taxon classificationAnimaliaColeopteraCarabidae

7297C7A08CE25121BDA33327F45FF95D

http://zoobank.org/341884A9-BC65-4443-B269-A962E3472D0A

#### Type material.

**Figures 26–27.**
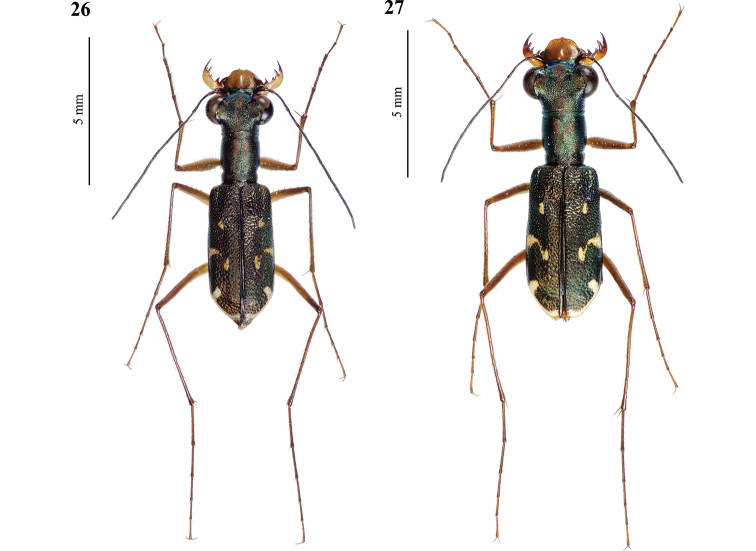
Type specimens of *Cylindera autumnalis* sp. nov. **26** male holotype (AdeC84-3) **27** female paratype (AdeC78-2).

***Holotype:*** male (Fig. 26; specimen code: AdeC84-3; dry pinned, with aedeagus in glycerol in a separated microvial labeled “AdeC84-3”): Taiwan, Pintung, Shuangliu Forest Recreation Area, 03 Sep. 2018, Ming-Hsun Chou leg. Original label: ”Locality: 屏東雙流森林遊樂區 / Date: 2018.IX.03 / Collector: 周明勳 / Code: AdeC84-3”; “NCHU 0011-0736”. Dry specimen and aedeagus of holotype deposited in NCHU. ***Paratypes***: 3 males (specimen code: AdeC48-4, AdeC48-5, and AdeC48-8, respectively; dry pinned, with aedeagus in glycerol in a separated microvial, respectively), 1 female (specimen code: AdeC48-1; dry pinned), and 1 female (specimen code: AdeC48-2; dry pinned, with genitalia preserved in glycerol in a separated microvial): Taiwan, Pintung, Shuangliu Forest Recreation Area, 10 Aug. 2017, Ming-Hsun Chou leg. 1 male (specimen code: AdeC78-1; dry pinned, with aedeagus in glycerol in a separated microvial) and 1 female (specimen code: AdeC78-2 (Fig. 27); dry pinned): Taiwan, Pintung, Shuangliu Forest Recreation Area, 21 Jul. 2018, Ming-Hsun Chou leg. 1 male (specimen code: AdeC84-1; dry pinned, with aedeagus in glycerol in a separated microvial) and 1 female (specimen code: AdeC84-2; dry pinned, with genitalia in glycerol in a separated microvial): same collecting information as for holotype. Original labels of paratypes see Table 2. All dry specimens and genitalia of paratypes deposited in NMNS.

#### Type locality.

Taiwan, Pintung, Shuangliu Forest Recreation Area.

#### Diagnosis.

**Figure 28.**
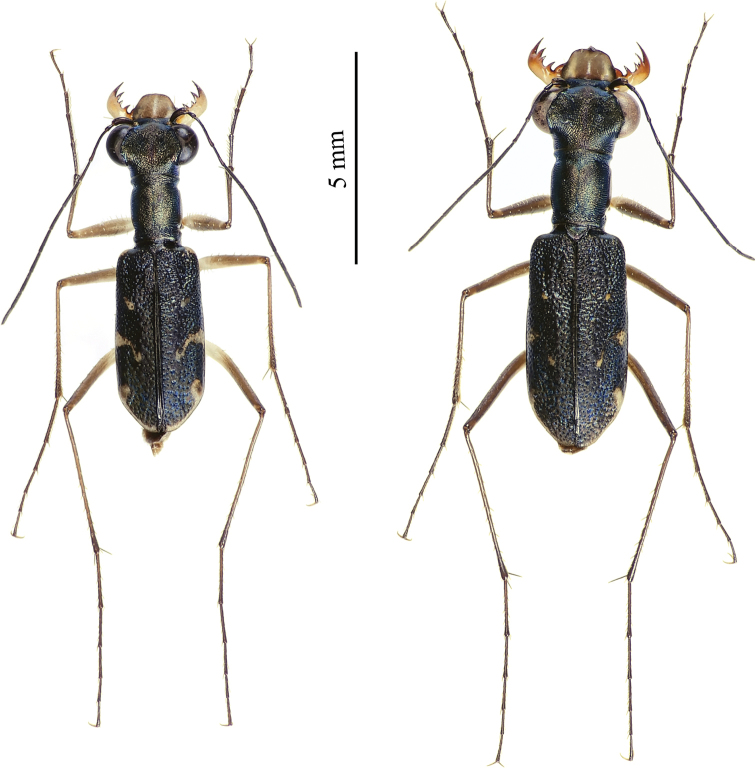
Dorsal habitus of *Cylindera pseudocylindriformis* (left – male; right – female).

**Figures 29–32.**
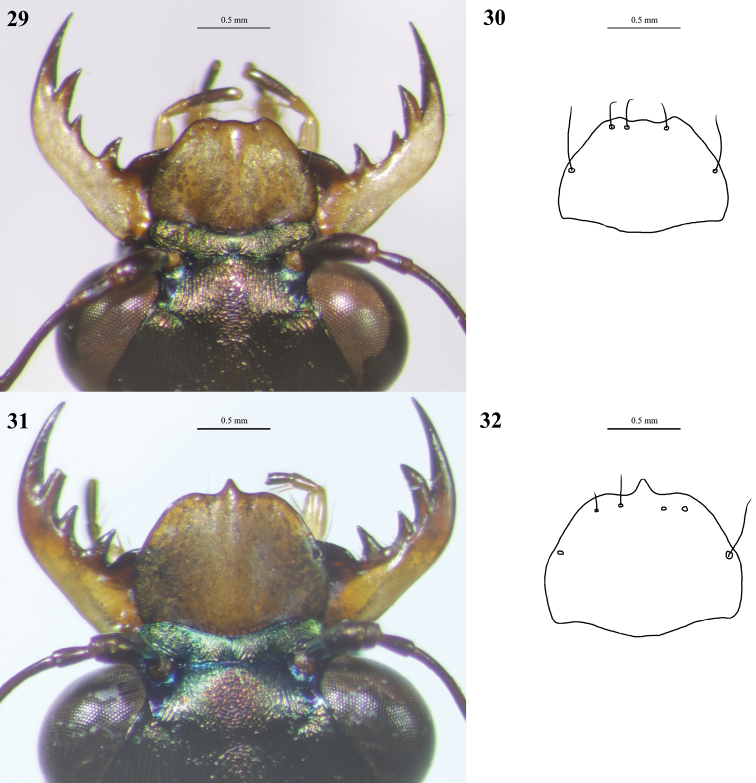
Digital images (left) and line drawings (right) of labra of *Cylindera autumnalis* sp. nov. **29, 30** male (holotype, AdeC84-3) **31, 32** female (paratype, AdeC78-2).

**Figures 33–36.**
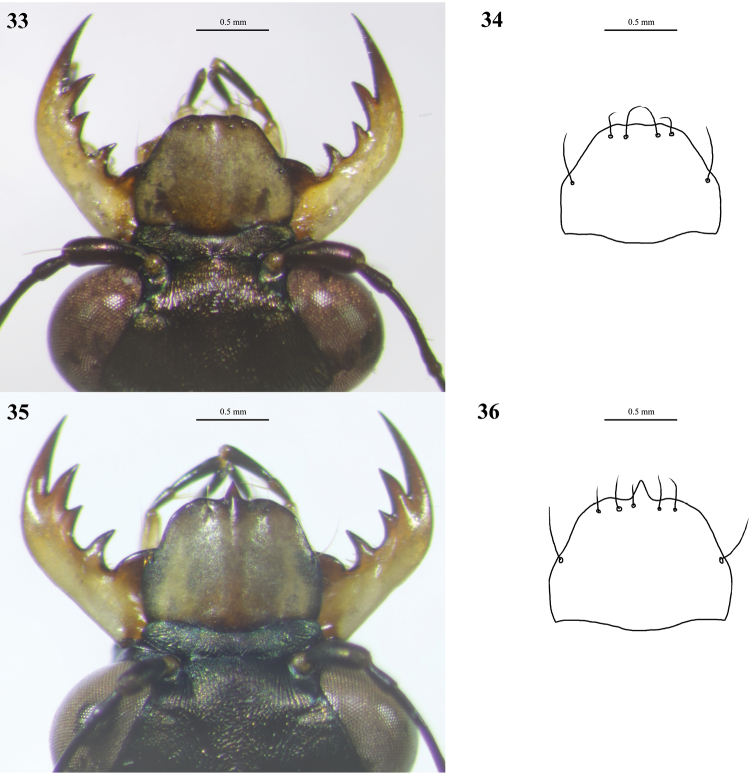
Digital images (left) and line drawings (right) of labra of *Cylindera pseudocylindriformis***33, 34** male **35, 36** female.

Elytra are metallic brownish and marked with obvious punctures. The apical lunula is thickened in both ends (subapical corner and apical end near suture). *Cylindera autumnalis* sp. nov. has a different body coloration and more obvious elytral maculation than *C. pseudocylindriformis* (Fig. 28). The former has few hairs on mesoepisterna in male and on metepisterna in both genders, but the latter’s mesoepisterna and metepisterna are glabrous in both genders. Body size of *C. autumnalis* sp. nov. male was significantly larger than *C. pseudocylindriformis* although is not statistically significant in female. Their labrum (Figs 29–32, 33–36) and male genitalia (Figs 12, 13) might be poorly distinctive.

#### Description.

***Head*** metallic bronze with weak greenish luster; genae dark metallic green; canthus with one seta; rugae longitudinal along frons, canthi, vertex, and lateral neck, and becoming transverse on genae; clypeus patina and microsculptured. ***Compound eyes*** large and protruding. ***Antennae*** slender and filiform; scape with one apical seta; 1–4 antennomeres metallic dark brown; 5–11 ones darker. ***Mandible*** yellowish pale with darker teeth, exceeding labrum when closed. ***Maxillary palps*** yellowish; last palpomere metallic dark testaceous. ***Labial palps*** yellowish; last palpomere metallic dark testaceous. ***Labrum*** testaceous; anterior margin rounded and unidentate in female; anterior margin without noticeable tooth or even concaved in male; margin with three or four preapical and two lateral setae (Figs 29–32). ***Pronotum*** cylindrical and metallic bronze with little greenish luster; dorsum transversely rugose; one transverse groove on each anterior and posterior dorsum portions, connected with one shallow longitudinal groove. ***Elytra*** bronze with metallic luster, slender, and marked with many obvious punctures; humeral spot present; posthumeral spot discoidal or irregular; one triangular spot on middle margin of elytron, connected with one clavate spot but disconnected in some individuals; apical lunula obvious, crescent; both subapical portion and apical end near suture of apical lunula thickened. ***Legs*** slender and testaceous, except metallic dark green coxae; some white hairs on coxae and femurs; pro-, mesocoxae, pro- and mesotrochanters with one long seta; pro-tarsi sexually dimorphic, basal 1–3 tarsomeres with short brush-like ventral setae and little wider than four or five tarsomeres in male, all pro-tarsomeres equivalent in width roughly and without brush-like ventral setae in female. ***Thoracic proepisternum*** dark metallic green, longitudinally rugose, and glabrous. ***Prosternum*** dark metallic green, transversally rugose, glabrous. ***Mesoepisternum*** dark metallic green, rugose and longitudinally depressed, with two or three hairs in male but glabrous in female. ***Mesosternum*** dark metallic green, transversally rugose, glabrous. ***Metepisternum*** dark metallic green, coarsely sculptured, with few hairs. ***Metasternum*** dark metallic green, microsculptured, and almost glabrous. ***Abdomen sternum*** dark green with little metallic luster, almost hairless, except one pair of long hairs on 4–6 segments. ***Aedeagus*** of holotype shown in Fig. 10. Description same as Results.

#### Etymology.

During the collection period in 2017 and 2018, this species was collected mostly in August to early September, especially in September. Many individuals could be found in early September when other tiger beetle adults disappeared mostly in that habitat. Thus, the specific name “*autumnalis*” means the autumnal tiger beetle.

#### Distribution.

Only known from type locality.

#### Ecology.

According to field observation, adults live in forest trails in late summer to autumn (late July to September). They crawl on the open ground and fly away for a short distance when being bothered, sometimes hiding in the grass or litters. The other two tiger beetle species which could be also found in the same habitat are *C. cylindriformis* and *Therates alboobliquatus alboobliquatus* Horn, 1909. However, adults of these three tiger beetles seem to appear in different seasons. *Cylindera cylindriformis* adults appear in early to mid-summer, and *T. a. alboobliquatus* was recorded mainly in mid-summer.

### Key to *Cylindera* species in Taiwan

**Table:** 

1	Labrum comparatively elongated (Figs 18–25, 29–36)	**2**
–	Labrum comparatively transverse (Figs 37–39)	**7**
2	Labrum tridentate	**3**
–	Labrum unidentate; anterior portion of labrum without obvious teeth or even concaved in male (Figs 29–30, 33–34)	**4**
3	Triangular spot on elytral middle edge present; subapical spot rounded or triangular	***C. sauteri***
–	Triangular spot on elytral middle edge absent; subapical spot elongated	***C. ooa* sp. nov.**
4	Labrum testaceous	**5**
–	Labrum not testaceous	**6**
5	Apical lunula linear and slender in apical end near suture; metepisternum without hairs; body color dark brownish or dark iron gray; elytral maculation sometimes obscure	***C. pseudocylindriformis***
–	Apical lunula thickened in apical end near suture; metepisternum with few hairs; body color metallic brownish; elytral maculation obvious	***C. autumnalis* sp. nov.**
6	Middle spot triangular and about half elytral width long	***C. cylindriformis***
–	Middle spot bended downward and more than half elytral width long	***C. redunculata***
7	Labrum tridentate (Fig. 39); posthumeral spot absent; body color brownish or iron gray	***C. shirakii***
–	Labrum unidentate (Figs 37, 38)	**8**
8	Posthumeral spot absent; underside covered by dense and long white hairs; body color gray or dark gray; elytral maculation usually tiny	***C. elisae reductelineata***
–	Posthumeral spot present	**9**
9	Body color brownish with green luster on head and pronotum; subapical spot oval or rounded and separated from apical spot; some individuals without apical spot	***C. psilica***
–	Not exactly fitting above description	**10**
10	Middle spot long, slender and bended down; underside covered by dense and long white hairs	***C. elisae formosana***
–	Elytral maculation varied, middle spot and apical lunula present, posthumeral spot ranging from tiny to large; labrum extended a little in anterior portion and with a small tooth in the middle of the extended portion; body color usually black gray but sometimes dark brownish	***C. kaleea***

**Figures 37–39.**
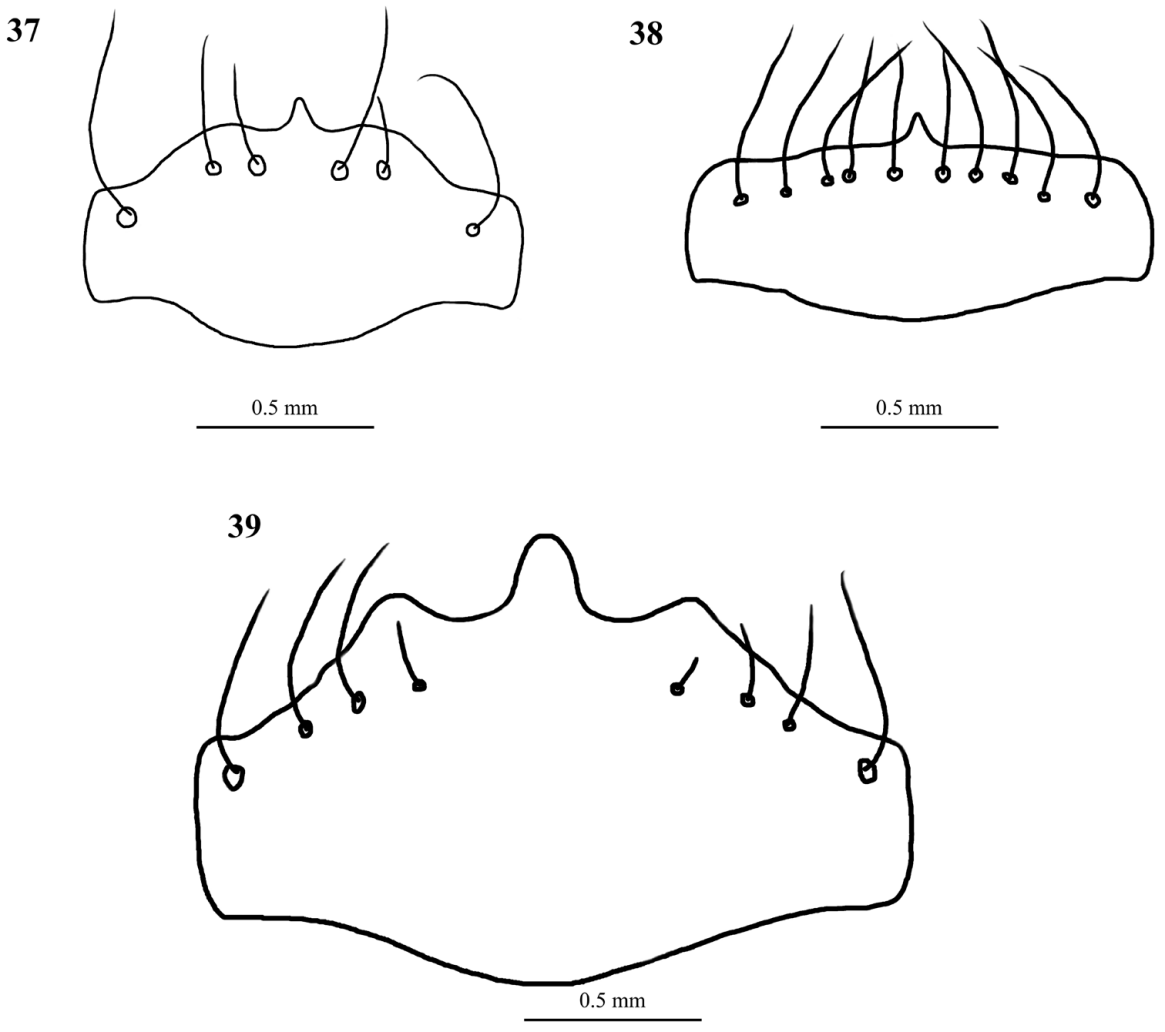
Line drawings of labra of Taiwanese *Cylindera* (female) **37***C. kaleea***38***C. elisae reductelineata***39***C. shirakii*.

## Supplementary Material

XML Treatment for Cylindera (Cylindera) ooa

XML Treatment for Cylindera (Cylindera) autumnalis

## Supplementary materials

Supplementary material 1 Tables S1–7 Data type: phylogenetic data Explanation note: **Table S1.** GenSeq and accession number of vouchered and type specimens. **Table S2.** Pairwise distances of COI of sauteri group. **Table S3.** Pairwise distances of 16S rDNA of sauteri group. **Table S4.** Pairwise distances of 28S rDNA of sauteri group. **Table S5.** Pairwise distances of COI of pseudocylindriformis group. **Table S6.** Pairwise distances of 16S rDNA of pseudocylindriformis group. **Table S7.** Pairwise distances of 28S rDNA of pseudocylindriformis group. https://binary.pensoft.net/file/339406This dataset is made available under the Open Database License (http://opendatacommons.org/licenses/odbl/1.0/). The Open Database License (ODbL) is a license agreement intended to allow users to freely share, modify, and use this Dataset while maintaining this same freedom for others, provided that the original source and author(s) are credited.Ming-Hsun Chou, Wen-Bin Yeh

Supplementary material 2 Figures S1–8 Data type: phylogenetic data Explanation note: **Figure S1.**ML tree inferred from COI of the sauteri group with ML bootstrap values (left) and BI posterior probability (right). **Figure S2.**ML tree inferred from 16S rDNA of the sauteri group with ML bootstrap values (left) and BI posterior probability (right). **Figure S3.**ML tree inferred from 28S rDNA of the sauteri group with ML bootstrap values (left) and BI posterior probability (right). **Figure S4.** Molecular clock inferred from the combined data of COI, 16S rDNA, and 28S rDNA of the sauteri group. **Figure S5.**ML tree inferred from COI of the pseudocylindriformis group with ML bootstrap values (left) and BI posterior probability (right). **Figure S6.**ML tree inferred from 16S rDNA of the pseudocylindriformis group with ML bootstrap values (left) and BI posterior probability (right). **Figure S7**ML tree inferred from 28S rDNA of the pseudocylindriformis group with ML bootstrap values (left) and BI posterior probability (right). **Figure S8.** Molecular clock inferred from the combined data of COI, 16S rDNA, and 28S rDNA of the pseudocylindriformis group. https://binary.pensoft.net/file/339407This dataset is made available under the Open Database License (http://opendatacommons.org/licenses/odbl/1.0/). The Open Database License (ODbL) is a license agreement intended to allow users to freely share, modify, and use this Dataset while maintaining this same freedom for others, provided that the original source and author(s) are credited.Ming-Hsun Chou, Wen-Bin Yeh
